# LC-MS/MS-based proteome profiling in *Daphnia pulex *and *Daphnia longicephala*: the *Daphnia pulex *genome database as a key for high throughput proteomics in *Daphnia*

**DOI:** 10.1186/1471-2164-10-171

**Published:** 2009-04-21

**Authors:** Thomas Fröhlich, Georg J Arnold, Rainer Fritsch, Tobias Mayr, Christian Laforsch

**Affiliations:** 1 Laboratory for Functional Genome Analysis (LAFUGA), Gene Center, Ludwig-Maximilian University, 81377 Munich, Germany; 2 Department of Biology II and GeoBio-Center, Ludwig-Maximilians-University Munich, 82152 Martinsried, Germany

## Abstract

**Background:**

Daphniids, commonly known as waterfleas, serve as important model systems for ecology, evolution and the environmental sciences. The sequencing and annotation of the *Daphnia pulex *genome both open future avenues of research on this model organism. As proteomics is not only essential to our understanding of cell function, and is also a powerful validation tool for predicted genes in genome annotation projects, a first proteomic dataset is presented in this article.

**Results:**

A comprehensive set of 701,274 peptide tandem-mass-spectra, derived from *Daphnia pulex*, was generated, which lead to the identification of 531 proteins. To measure the impact of the *Daphnia pulex *filtered models database for mass spectrometry based *Daphnia *protein identification, this result was compared with results obtained with the Swiss-Prot and the *Drosophila melanogaster *database. To further validate the utility of the *Daphnia pulex *database for research on other *Daphnia *species, additional 407,778 peptide tandem-mass-spectra, obtained from *Daphnia longicephala*, were generated and evaluated, leading to the identification of 317 proteins.

**Conclusion:**

Peptides identified in our approach provide the first experimental evidence for the translation of a broad variety of predicted coding regions within the *Daphnia *genome. Furthermore it could be demonstrated that identification of *Daphnia longicephala *proteins using the *Daphnia pulex *protein database is feasible but shows a slightly reduced identification rate. Data provided in this article clearly demonstrates that the *Daphnia *genome database is the key for mass spectrometry based high throughput proteomics in *Daphnia*.

## Background

During the last two decades, genome sequencing efforts are providing us with complete genome sequences from many organisms (for a summary refer to ). The generated sequence databases are fundamental tools used by researchers in almost every field of modern biology. In addition they provide the basis for powerful technologies to quantitatively analyze the gene expression profile on the mRNA-level using DNA microarrays [1,2]. However, it has to be considered that mRNA molecules are only intermediate products towards the production of functional proteins and that protein abundance is not necessarily reflected by the amount of the corresponding mRNA transcript [3,4]. The concentration of individual proteins at the cellular level or in biological fluids mainly depends on four completely different processes: (i) protein synthesis, (ii) protein processing, (iii) protein secretion and (iv) protein degradation. As a consequence, systematic quantitative predictions of protein populations are impossible to deduce from genomic or transcriptional data. Moreover, proteins frequently undergo post-translational modifications (PTMs) crucial for their function, activity, and stability and they often play major roles in regulatory networks [5]. Comprehensive datasets addressing the protein level, therefore, are indispensable for a functional and biochemical characterization of both cells and organisms. The field of high-throughput identification and quantification of proteins using systematic approaches is commonly referred to as proteomics. Recent developments in mass spectrometry have revolutionized the field and dramatically increased the sensitivity of protein identification compared to classical techniques like Edman sequencing. As a consequence, large proteome investigations have been established covering, e.g., human plasma [6], human brain [7] and human liver [8] as well as model organisms such as *Caenorhabditis elegans *[9] and *Drosophila melanogaster *[10].

This, in turn, has led to the realization that proteomics is not only essential to our understanding of cell function, but in addition is a validation tool for genes predicted in genome annotation projects. Recently published results demonstrate that peptide mass spectrometry complements gene annotation in *Drosophila *[10] and humans [11,12].

Although a multitude of whole-genome sequencing projects ranging from microbial (e.g. [13]) to vertebrate genomes [14] have been initiated in the last decade, no complete genome sequence is available for crustaceans, a species-rich taxa with additional high economical impact.

Hence, the *Daphnia *Genomics Consortium (DGC; ) was founded in 2003 to develop the waterflea *Daphnia*, a small planktonic crustacean, as a further model system in genomics, but with the added advantage of being able to interpret the results in the context of natural ecological challenges. Even though the ecology and ecotoxicology of *Daphnia *has been well studied, because they are a major link between limnetic primary production and higher trophic levels, less work has been done on the genetics of this organism. Nevertheless, their clonal reproduction, short generation times, and their transparent body also make them well suited for experimental molecular research.

In this special series of papers published in BMC journals, the first description of the *Daphnia pulex *draft genome sequence  is described. Besides investigation on the DNA and mRNA level, the availability of the *Daphnia *genome sequence opens the door to investigate the proteome of this fascinating species. In this article we present the generation of a first data-set consisting of 701,274 peptide tandem-mass-spectra derived from *Daphnia pulex*. In order to demonstrate the impact of the *Daphnia *genome sequence on proteomics based studies we compared the number of identified proteins using the *Daphnia *protein database with the number of identifications obtained by searching against the Swiss-Prot and the *Drosophila melanogaster *protein database . To validate the utility of the *Daphnia pulex *genome for research on different *Daphnia *species, additional 407,778 peptide tandem-mass-spectra derived from *Daphnia longicephala *were generated and evaluated. In addition, the peptides identified in our approach provide the first experimental evidence for the translation of a broad variety of predicted coding regions within the *Daphnia *genome.

## Results

### Sample preparation

To generate protein lysates suitable for SDS gel electrophoresis, pools of about 300 waterfleas (*Daphnia pulex *and *Daphnia longicelphala *respectively) were homogenated. The protein concentration of the obtained lysates (2 mL) was 2.6 mg/mL for *Daphnia pulex *and 2.3 mg/mL for *Daphnia longicephala *corresponding to a total protein yield of 17 μg and 15 μg per Daphniid, respectively.

### SDS-gel pre-fractionation of *Daphnia proteins*

**Figure 1:**
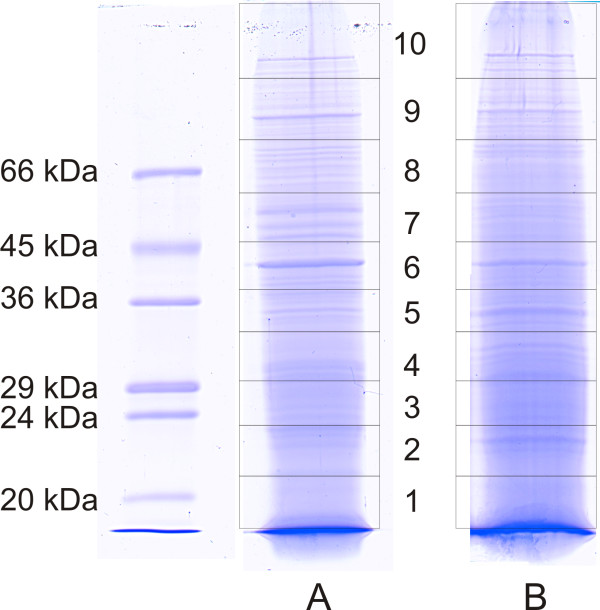
**Coomassie stained SDS-gels of *Daphnia *proteins**. Scanned image of Coomassie stained SDS-gels. Panel A: 50 μg *Daphnia pulex *protein; Panel B: 50 μg *Daphnia longicephala *protein. The rectangles indicate the 10 gel slices which were individually analyzed by LC-MS/MS.

50 μg of total protein from either *Daphnia pulex *or *Daphnia longicephala*, was separated by SDS-gel electrophoresis. To evaluate the quality of the electrophoretic separation, the gels were stained with Coomassie. An image of SDS-gels derived from both *Daphnia *species is shown in Fig. 1. Both samples showed sharp distinct bands, indicating that the performed electrophoreses had good separation strengths. To generate 10 protein fractions of each sample, the corresponding gel lanes were cut into 10 pieces as outlined in Fig. 1. To get samples suitable for LC-MS/MS, each gel slice was subjected to the in-gel digestion procedure described in the Methods chapter.

### LC-MS/MS analysis of *Daphnia pulex *proteins

**Figure 2:**
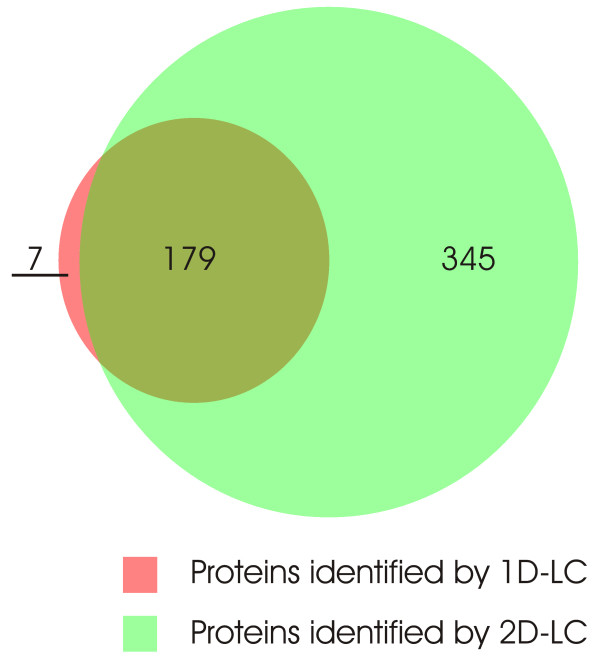
**Proportional Venn diagram of protein overlap**. Proportional Venn diagram demonstrating the degree of overlap of proteins identified by 1D-LC-MS/MS and 2D-LC-MS/MS.

**Figure 3:**
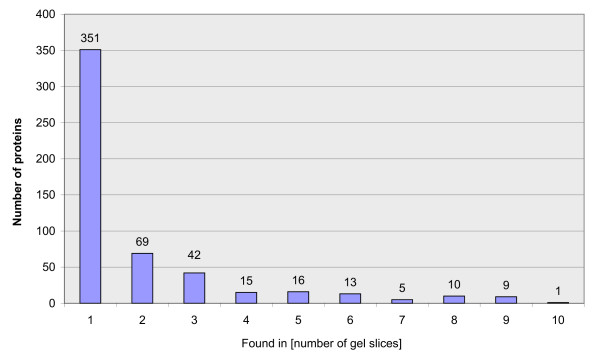
**Identified proteins**. Bar chart indicating the number of proteins identified in more than one gel slice. X axis number of gel slices, Y axis number of identified proteins by 2D-LC-MS/MS.

For the qualitative analysis of the *Daphnia pulex *proteome, two samples were fractionized by SDS-gel electrophoresis (as described in the above paragraph) and subjected to LC-MS/MS analysis. Each of the 10 gel fractions was separated with one-dimensional reversed phase (RP) liquid chromatography (1D-LC) and a combination of strong cation-exchange (SCX) with RP chromatography (2D-LC) respectively. From the 1D-LC-MS/MS runs 100,462 spectra could be collected and from the 2D-LC-MS/MS runs 600,812 spectra were acquired. All MS/MS spectra were searched against the non-redundant filtered models database of *Daphnia *v1.1 gene builds (July, 2007)  and evaluated using the PeptideProphet software. Applying a false discovery rate of = 1%, 7973 MS/MS spectra could be assigned to peptides within the *Daphnia *database, of which 1654 were unique. The assignment of peptides to proteins using the ProteinProphet algorithm led to the identification of 186 proteins with the 1D-LC-MS/MS approach and 524 proteins with the 2D-LC-MS/MS startegy (false positive discovery rate = 1%). As shown in Fig. 2, all except seven proteins identified in the 1D-LC approach could be found in the 2D-LC-MS/MS dataset as well. Further analysis of the data revealed that a significant fraction of proteins could be identified in more than one gel slice, as summarized in Fig. 3. The overall list of identified proteins and peptides is available as additional file 1.

### Ontology analysis of the identified proteins

**Figure 4:**
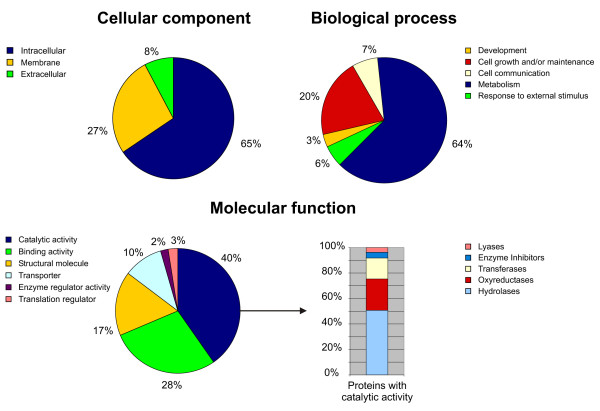
**Ontology analysis**. Ontology analysis of 501 identified *Daphnia pulex *proteins. The classification of the protein set was performed according to the gene ontology terms: "Cellular component", "Biological process" and "Molecular function".

To analyze the ontology of the identified *Daphnia pulex *proteins the entries of the filtered models database were BLASTp-searched  in the Swiss-Prot database [15]. We chose the Swiss-Prot database because of its high level of annotation, including entries about protein function, posttranslational modifications as well as a direct link to the Gene Ontology (GO) databases [16]. From the 531 sequences derived from the filtered models database, 499 homologue (E-values < 0.01) protein sequences could be found. The corresponding protein Swiss-Prot IDs were subjected to ontology analysis using the PANDORA server . The results of this ontology analysis are shown in Fig. 4. In the "cellular component" GO database only 139 proteins of the 499 proteins were listed. Their classification analysis revealed that the majority (65%) are of intracellular origin and the fraction of the particularly interesting class of membrane proteins comprises 27%. The "molecular function" GO revealed 350 proteins the majority of which were classified as proteins with catalytic activity. From these fractions 141 were enzymes from which 68 could be classified as hydrolases, 33 as oxyreductases, 22 as transferases and 5 as lyases. 6 proteins could be classified as enzyme inhibitors. Using the "biological process" database 272 proteins could be classified from which 175 were associated with metabolism, 55 with cell growth and/or maintenance, 18 with cell communication, 15 with response to external stimulus and 9 with developmental processes.

### Searches of MS/MS data in the Swiss-Prot and *Drosophila melanogaster *protein database

To investigate the benefit of the *Daphnia pulex *filtered models database on the MS based identification of *Daphnia *proteins, cross-species identification, as suggested by several authors [17,18], was performed using the *Metazoa *subset of the Swiss-Prot database (Release 54.2, 78,385 entries) and the *Drosophila melanogaster *database from FlyBase (20,726 entries). Using the MS/MS spectra obtained with the 2D-LC-MS/MS runs of the *Daphnia pulex *sample, 71 *Daphnia *proteins could be identified with the *Drosophila *database and 92 with the Swiss-Prot database with a false-positive identification threshold of = 1%.

### LC-MS/MS analysis of *Daphnia longicephala proteins*

**Figure 5:**
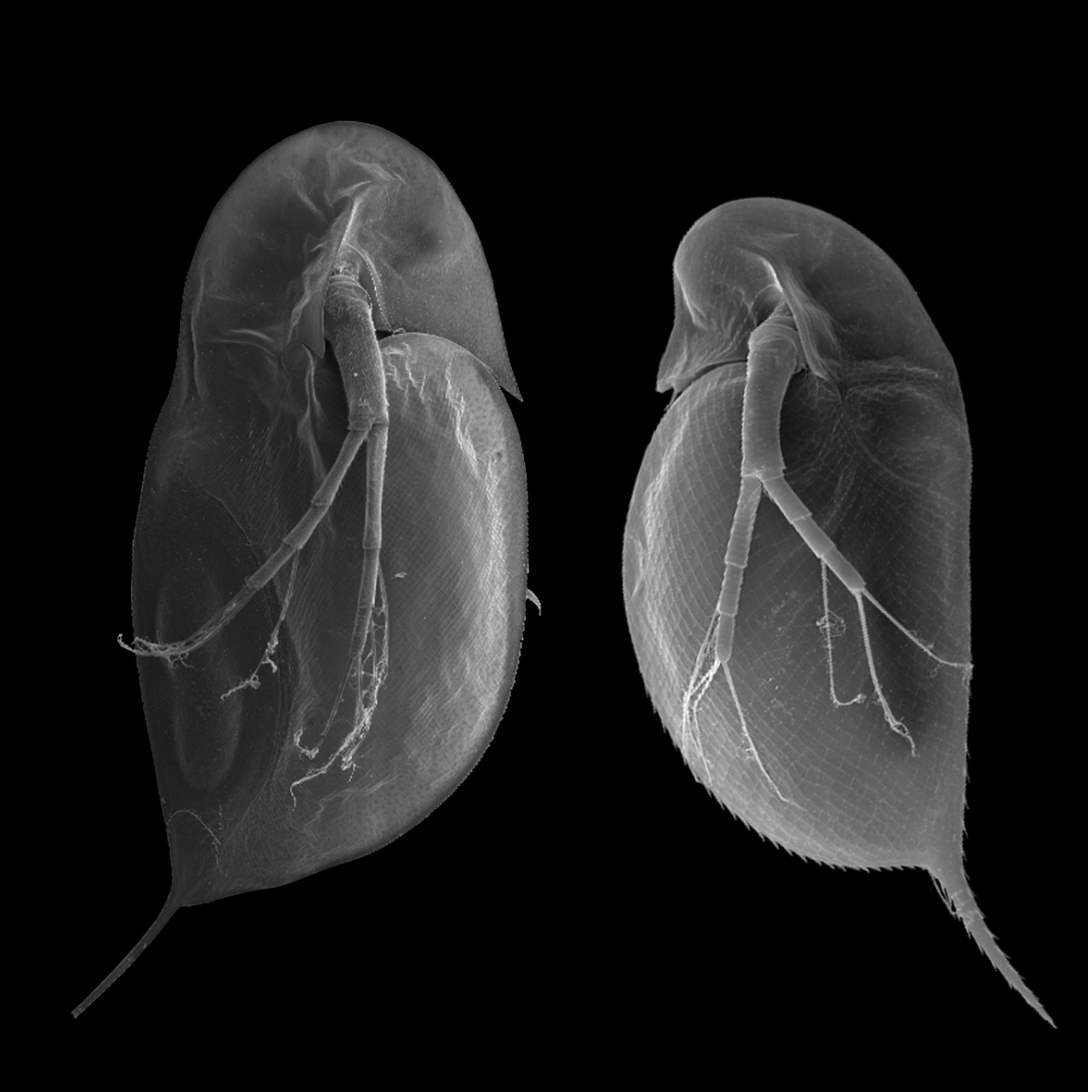
***Daphnia *images**. Scanning electron micrograph from *Daphnia longicephala *(l) and *Daphnia pulex *(r).

**Figure 6:**
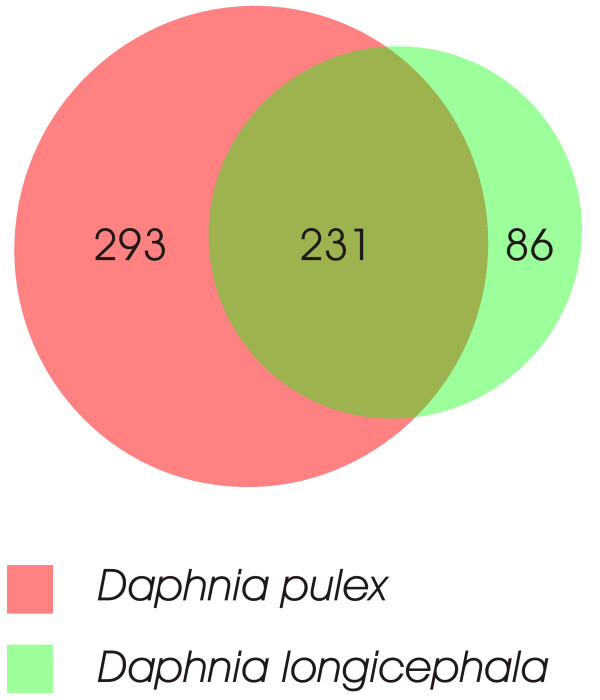
**Proportional Venn diagram of protein overlap**. Proportional Venn diagram demonstrating the overlap of proteins identified from *Daphnia pulex *and *Daphnia longicephala*.

To determine the suitability of the non-redundant filtered models database of putative *Daphnia pulex *proteins for the MS-based identification of proteins from other *Daphnia *subgenera, a *Daphnia longicephala *protein lysate was generated. (A scanning electron micrograph from both, *Daphnia pulex *and *Daphnia longicephala *is shown in Fig. 5. For the protein identification exactly the same separation strategy as for *D. pulex *was used. Using this SDS-PAGE – 2D-LC-MS/MS combination and the non-redundant filtered models database of putative *Daphnia pulex *proteins, we were able to identify 671 unique peptides (PeptideProphet, false discovery rate = 1%) which could be assigned to 317 *Daphnia longicephala *proteins (ProteinProphet, false discovery rate = 1%). As shown in Fig. 6, 86 of these proteins could exclusively be identified in *Daphnia longicephala *samples but not in *Daphnia pulex *samples.

## Discussion

### General remarks

For a comprehensive functional and biochemical characterization of organisms, an inventory of their proteins and protein modifications is a prerequisite. In the work presented here, we performed a liquid chromatography – mass spectrometry based qualitative proteome approach with the goal to generate a first protein catalogue of *Daphnia pulex*, the genome of which is presented in this special issue. To complement gene sequences, the generation of a broad dataset of tandem MS (MS/MS) spectra derived from *Daphnia *peptides is particularly interesting for two main reasons:

i) High throughput MS/MS protein identification is based on the comparison of experimentally acquired peptide MS/MS spectra with *in silico *generated theoretical spectra deduced from protein databases. With a large set of MS/MS spectra it can be tested if the *Daphnia *filtered models protein database is suitable in its current form for proteomics approaches, which are mostly based on protein identification by MS/MS.

ii) The generation of MS/MS spectra derived from *Daphnia *peptides will lead to the creation of a catalogue of identified daphniid peptides. This will be one of the first datasets giving experimental evidence for a variety of so far only predicted proteins. The *Daphnia *filtered models protein database in its current form consists of more than 30,000 entries. The corresponding genes were either found by EST sequencing, by homology searches, or *ab initio *by gene prediction algorithms. However, for the broad majority of database entries, there is so far no experimental evidence that the corresponding genes are in fact translated and the resulting proteins persist in the organism.

### Experimental strategy

Among all presently available proteomic techniques, the application of liquid chromatography (LC) as a separation tool combined with electrospray ionization (ESI) [19] tandem mass spectrometry (MS/MS) as an identification tool has the highest performance in terms of protein identifications per time unit. This technique is referred to as LC-MS/MS and has proven its efficiency in many studies [20-22]. Since eukaryotic proteomes consist of highly complex mixtures, the reduction of complexity by pre-fractionation on the level of intact proteins prior to LC-MS/MS analysis is mandatory. The number of identifications usually increases with the overall extent of prefractionation efforts. Because of its high separation strength we choose 1D-SDS-gel electrophoresis for pre-fractionation on the protein level. In this pilot study a number of 10 gel fractions were chosen. To determine the impact of two versus one chromatographic steps on the number of identified peptides, we compared the results obtained with one-dimensional reversed phase (RP) liquid chromatography (1D-LC) versus a combination of strong cation-exchange (SCX) with RP chromatography. The major advantage of the SCX – RP combination is the removal of salt ions from the SCX fractions in the RP step, which would otherwise interfere with the MS-analysis of peptide ions. For reasons of performance, we choose a fully automatic online setup, where SCX fractions are directly eluted onto a RP trap column. This RP trap column is then switched into the RP chromatography system to finally separate the peptides. The SCX flow through as well as 6 salt fractions from each of the 10 gel slices were captured and analyzed by LC-MS/MS; leading to a total number of 80 1D-LC-MS/MS runs (10 gel slices × 1 RP-LC run + 10 gel slices × 7 SCX fractions × 1 run 1 RP-LC run). From this workflow, 701,274 MS/MS spectra were obtained.

### Results obtained with LC-MS/MS

Using SDS-PAGE combined with 1D-LC-MS/MS, we identified 186 entries whereas the SDS-PAGE – 2D-LC-MS combination led to the identification of 524 entries from the non-redundant filtered models database of putative *Daphnia *proteins demonstrating the benefit of a second chromatographic step. In total, we were able to identify 531 non-redundant filtered models database proteins of putative *Daphnia pulex *proteins. The overall list of identified proteins can be downloaded as additional file 1.

Considering that the main goal of our experiments was to test the benefit of a dedicated *Daphnia *protein database for LC-MS/MS-based proteomics, this result is promising with respect to the straightforward design of this pilot study. As recently demonstrated by [10], an extensive prefractionation on the level of the biological sample (e.g. selection of different development stages), on the cellular, on the subcellular level as well as on the level of proteins and peptides had to be performed to get a catalogue of thousands of experimentally identified proteins from *Drosophila*. Our results clearly demonstrate that LC-MS/MS analysis combined with the usage of the *Daphnia *filtered models database is able to identify hundreds of *Daphnia *proteins with a high confidence level in a very efficient way. Therefore, this methodology combined with further pre-fractionation steps will lead to an increased analytical depth of the *Daphnia *proteome.

### Determination of false positive ratios

The general strategy to identify peptides by high-throughput MS/MS experiments is a probability based comparison of experimental spectra with theoretical spectra calculated from protein databases deduced from DNA sequences. The software algorithms determine the closest match and a score indicating the reliability of the result. Although this identification strategy has proven its strength in many studies, cut-off values for the obtained scores must be chosen carefully to minimize false-positive identifications [23,24]. Unfortunately, there are no general rules for the confidence of given scores, because their reliability depends on the experimental setup as well as on the database used for the search. In our study, we applied the commonly used Mascot [25] search engine, returning a so called "ions score" for each peptide (for details see . However, special care must be taken when peptides spectra are used as evidence for the existence of corresponding proteins. Since a given peptide sequence can be present in multiple proteins, these shared peptides can lead to an overestimation of the number of identified proteins as well as to an under-estimation of the false discovery rate. An overview of this issue was given by Nesvizhskii et al. [26]. Therefore, to validate the Mascot search results we used the Trans-Proteomic Pipeline [27] downloadable from the Seattle Proteome Center . This software package includes PeptideProphet  to compute probabilities for identified peptides [28] and ProteinProphet  to address the issue of shared peptides and to calculate the probabilities of corresponding protein identifications [29]. To further confirm the false positive ratio given by the Trans-Proteomic pipeline we generated a so-called decoy version of the *Daphnia pulex *filtered models database consisting of random sequences with the same average amino acid composition. This decoy database was attached to the original database and then used to search our MS/MS spectra as proposed by Elias et al. [30]. Any protein hit derived from the decoy part of the combined database was regarded as false-positive identification. The number of four hits from the decoy part of the database is in accordance with the 1% false discovery rate calculated by the Trans-Proteomic Pipeline.

### Proteolytic activity

The analysis of the data revealed that a significant fraction (34%) of proteins could be identified in more than one gel slice, as summarized in Fig. 3. A heterogeneity of molecular masses is frequently observed in this kind of approaches [31,32]. and may be caused by posttranscriptional events such as alternative splicing, posttranslational modifications or proteolytic processing. While, inadequate separation strength of the gel can be excluded due to the presence of sharp distinct bands (see Fig. 1), proteolysis of these proteins prior to electrophoresis may contribute to this heterogeneity. Proteolysis can be caused by *Daphnia *proteases from the intestinal tract. The proteolytic activity of *Daphnia magna *gut protease was previously described [33,34]. In preliminary studies in which we performed 2D-gel electrophoresis of *Daphnia magna *and *Daphnia longicephala *lysates, we tried to eliminate this proteolytic activity with several commercially available protease inhibitor cocktails. The list of tested inhibitors, including the used concentrations, is shown in Table 1. However, the obtained spot patterns of all prepared 2D-gels still reflected significant protein degradation (Data not shown).

**Table 1:** List of tested protease inhibitors

**Protease Inhibitor**	**Concentration**
Protease Inhibitor Cocktail P8340 (Sigma Aldrich)	2%

Protease Inhibitor Cocktail P2714 (Sigma Aldrich)	3%

Complete Protease Inhibitor Cocktail Tablets (Roche)	1 tablet in 25 mL extraction buffer

APMSF, TLCK, Chymostatin (Sigma Aldrich)	79 μM; 160 μM; 83 μM

TLCK, TPCK (Sigma Aldrich)	160 μM; 143 μM

Soybean Trypsin Inhibitor T 9003 (Sigma Aldrich)	250 μM

As the efficient inhibition of *Daphnia *proteases plays a crucial role in further quantitative proteome studies, we screened our catalogue of identified *Daphnia *proteins for proteases. In total, we have identified 19 different proteins out of the *Daphnia *database showing significant homology (BLAST E-value < 0.01) to known proteases with exo- as well as endopepdidase activity (Table 2). In the case of the *Daphnia *trypsin proteases identified, the masses of the detected peptides did not fit with the theoretical peptide masses of the porcine trypsin used for digestion of the samples. Hence, these peptides clearly originate from *Daphnia *proteins. The list of *Daphnia *proteases in Table 2 provides a basis for further sophisticated experiments, e.g. determination of cleavage specificities and screening for protease inhibitors.

**Table 2:** List of identified *Daphnia *proteins homologue to proteins with known exo- or endopeptidase activity

**Dappu Protein ID**	**Homolgue to [Swiss-Prot Accession]**	**E-Value**	**Protein name**	**Activity**
302098	P13676	9E-109	Acylamino-acid-releasing enzyme (EC 3.4.19.1) (Acyl-peptide hydrolase) (APH) (Acylaminoacyl-peptidase)	acylaminoacyl-peptidase activity

203795	P15145	7E-155	Aminopeptidase N (EC 3.4.11.2) (pAPN) (Alanyl aminopeptidase) (Microsomal aminopeptidase) (Aminopeptidase M) (CD13) (gp130)	Aminopeptidase activity

301437	Q27245	2E-103	Putative aminopeptidase W07G4.4 in chromosome V (EC 3.4.11.-)	aminopeptidase activity

307838	Q10751	0.0	Angiotensin-converting enzyme (EC 3.4.15.1) (Dipeptidyl carboxypeptidase I) (Kininase II) (Fragment)	carboxypeptidase activity

195011	P04069	1E-90	Carboxypeptidase B (EC 3.4.17.2)	carboxypeptidase activity

300872	Q95029	3E-123	Cathepsin L precursor (EC 3.4.22.15) (Cysteine proteinase 1)	cysteine-type endopeptidase activity

230618	Q86GF7	1E-36	Crustapain	cysteine-type endopeptidase activity

126867	P12955	5E-166	Xaa-Pro dipeptidase (EC 3.4.13.9) (X-Pro dipeptidase) (Proline dipeptidase) (Prolidase) (Imidodipeptidase)	metallocarboxypeptidase D activity

201234	Q80W54	3E-135	Farnesylated-proteins converting enzyme-1	metalloendopeptidase activity

200339	P23004	2E-66	Ubiquinol-cytochrome C reductase complex core protein 2, mitochondrial precursor (EC 1.10.2.2) (Complex III subunit II)	metalloendopeptidase activity

200882	O16796	3E-141	Neprilysin-2	Probable cell surface protease

231027	P00771	2E-62	Brachyurin (EC 3.4.21.32) (Collagenolytic protease)	serine-type endopeptidase activity

230885	P00765	1E-52	Trypsin I (EC 3.4.21.4)	serine-type endopeptidase activity

26258	Q00871	3E-52	Chymotrypsin BI precursor (EC 3.4.21.1)	serine-type endopeptidase activity

230174	P36178	3E-49	Chymotrypsin BII precursor (EC 3.4.21.1)	serine-type endopeptidase activity

231152	P49275	9E-36	Mite allergen Der f 3 precursor (EC 3.4.21.-) (Der f III)	serine-type endopeptidase activity

231560	P04814	3E-21	Trypsin alpha precursor (EC 3.4.21.4)	serine-type endopeptidase activity

307138	P97321	3E-119	Seprase (EC 3.4.21.-) (Fibroblast activation protein alpha) (Integral membrane serine protease)	serine-type endopeptidase activity; exopeptidase activity
303911	Q9D1A2	2E-178	Cytosolic non-specific dipeptidase (CNDP dipeptidase 2) (Glutamate carboxypeptidase-like protein 1)	Metallopeptidase activity

### Usability of the *D. pulex *filtered models database for proteome research on other *Daphnia *subgenera

In phylogenetics, the genus *Daphnia *is split into three subgenera, *Daphnia*, *Hyalodaphnia *and *Ctenodaphnia*. Sequence divergence between those subgenera indicates an origin in the Mesozoic [35]. Evolution under different environmental conditions such as UV radiation, salinity or predator regimes was certainly a key factor for diversification in this genus. To validate the utility of the *Daphnia pulex *genome sequence for proteome research on differing *Daphnia *species, we generated LC-MS/MS data of *D. longicephala *samples. *D. longicephala *was chosen due to the fact that it belongs to the taxon of *Ctenodaphnia*, in contrast to *D. pulex *which is grouped in the subgenus *Daphnia*. Moreover, *D. longicephala *is one of the most prominent examples for morphological plasticity [36] and provides an ideal model organism for future work on the genetic basis of the phenomenon of phenotypic plasticity.

For the proteome analysis of *D. longicephala*, identical amounts of total protein and the same 2D-LC-MS/MS strategy outlined for *D. pulex *was used. We were able to identify 317 proteins from the non-redundant filtered models database of putative *Daphnia pulex *proteins. The difference in number of identified proteins in *D. pulex *(524 in 2D-LC-MS/MS) may well mirror the genetic divergence between both *Daphnia *subgenera. This finding reflects the fact that even a single amino acid exchange in a given peptide mostly impairs its automatic identification by MS/MS search algorithms. Nevertheless, the number of identifications obtained from *D. longicephala *samples demonstrates the suitability of the *D. pulex *filtered models database for proteome investigations with other *Daphnia *subgenera.

Another finding is that 86 proteins were exclusively found in the *Daphnia longicepha *samples as illustrated in Fig. 6. This result might reflect different concentrations of a given protein in lysates of D. *pulex *and D. *longicephala*, e.g. through different metabolic activity and/or differences in their cellular assembly. On the other hand, this result may be due to undersampling, i.e., in highly complex samples, the number of co-eluting peptides exceeds the number of MS/MS spectra which can be acquired by the instrument. Therefore in individual LC-MS/MS runs, different low-intensity peptides may be selected for MS/MS analysis by the instrument software. The overall list of identified proteins can be downloaded as additional file 2.

### The impact of the *D. pulex *filtered models database for proteome research of Daphniids

Although several genome projects on crustaceans are in progress, only expressed sequence tag (EST) libraries (e.g. [37]) or the sequence of the mitochondrial genome [38] are available in other crustacean species. In cases where only few protein sequences are known, it is a common strategy to search MS/MS-data against databases of the most related species in order to identify identical peptides within the homologous proteins.

To estimate the impact of the *D. pulex *filtered models database for high-throughput proteomics of Daphniids, we compared the results obtained with the *Daphnia *database with the results obtained by searching our MS/MS dataset against two additional databases: As a species specific database we selected the *Drosophila melanogaster *database from FlyBase [39] (Release 5.2; ) consisting of 20,726 protein sequences. We chose this species because *D. melanogaster*, belongs to the taxon of *Hexapoda *(*Insecta *and relatives) and is the closest relative of *Daphnia pulex *with a characterized complete genome sequence [40]. Both arthropod species belong to a group called *Pancrustacea*, although monophyly of this group is still discussed [41].

The Pancrustacean hypothesis, which is supported by molecular analysis (e.g. [42]), queries that Myriapoda are the closest relatives to Hexapoda but renders crustaceans and hexapods as sister taxa. Given that the latter have likely diverged 550 to 650 million years ago [43] and have evolved in completely different habitats – crustaceans predominantly in aquatic, insects in terrestrial environments – it is expected that protein expression should reflect these evolutionary challenges. Even though some crustacean gene families, such as genes responsible for embryonic development are shared with Hexapoda [44], several *Daphnia *genes show no sequence similarity to other arthropods [45]. Therefore, gene transcripts different from those of *D. melanogaster *might reflect adaptations to aquatic habitats such as chemoreception, oxygen uptake or osmoregulation.

As a protein database of a broad variety of species we chose the *Metazoa *subset of the Swiss-Prot database (Release 54.2, 78,385 entries) providing a minimum of redundancy. To facilitate a comparison of the results obtained with the different databases, searches of MS/MS spectra were performed using exactly the same parameters. Setting a false-positive identification threshold of 1%, only 71 *Daphnia *proteins matched to the *Drosophila *database and 92 to the Swiss-Prot database. This finding clearly demonstrates that the *D. pulex *filtered models database in its current form increases dramatically the number of MS-based identifications and represents an indispensable tool for high-throughput proteome experiments in daphniids. However, many proteins may still be missing in the database. Therefore, yet unassigned spectra in our data set can help to find undisclosed coding regions within the *Daphnia *genome. Suitable algorithms comprise searching against the entire *Daphnia *genome sequence or *de-novo *sequencing – MS BLAST approaches as described by Shevchenko et al. [46]. Finally, the database supports detailed 2D gel analyses to quantify and identify proteins. The application of the latter technique allows the determination of isolelectric points and molecular weights of the proteins and enables the detection of protein isoforms by comparison of experimentally determined IPs with theoretical IPs from database analysis.

## Conclusion

Given that *Daphnia *is an important model organism, for instance to test for deleterious effects of pollutants or environmental changes, the implementation of state of the art techniques in molecular biology such as LC-MS/MS is an auspicious opportunity to unravel mechanisms triggering those critical environmental issues.

Our study is the first applying a LC-MS/MS based proteomic approach in *Daphnia *that reflects the utility of the *Daphnia *genome database for molecular works on this multifaceted model organism in several fields of biological research. Since a variety of *Daphnia *species are used for different scientific approaches, for instance to elucidate the phenomenon of phenotypic plasticity in daphniids [47] at least 20 species have been investigated intensively, it is essential to know the reliability of the *Daphnia pulex *genome sequence for studies on other species. We give experimental evidence for the translation of a broad variety of predicted coding regions within the *Daphnia *genome by using high throughput MS/MS protein identification in two *Daphnia *species. Our data demonstrates the applicability of proteomics research in *D. pulex *as well as in other *Daphnia *species. This will stimulate work on hypothetical functions for yet unclassified proteins followed by functional experiments in this new model organism. Moreover, proteomics techniques allow to identify proteins linked to biological phenomena such as induced predator defenses, host parasite-interactions or stress responses to toxic substances.

## Methods

### *Daphnia *cultures

We used a laboratory-cultured clonal line of *Daphnia pulex *and *Daphnia longicephala *for our experiments. The *Daphnia pulex *clone "The Chosen One" picked by the *Daphnia Genomics Consortium *for the sequencing project was isolated from an ephemeral pond in Oregon (USA) whereas *Daphnia longicephala *was isolated from Lara Pond (Australia).

Age-synchronized cohorts of both *Daphnia *species were grown prior to the experiments by collecting mothers with freshly deposited eggs. We cultured the latter in 30 L plastic buckets in the laboratory under constant conditions in a temperature-controlled room at 20°C ± 0.5. Fluorescent light was used to simulate a day-night rhythm (16 h day: 8 h night). The daphnids were fed daily with *Scenedesmus obliquus *at a concentration of 1.5 mg C L-1 to avoid food limitation. A synthetic medium based on ultra-pure water, trace-elements and phosphate buffer, was changed weekly [48]. 300 randomly chosen adult daphnids were collected prior to proteome analysis.

### Sample preparation

The medium containing the daphnids was filtered through a fine sieve (mesh aperture 125 μm) and immediately grounded in a pre-cooled ceramic mortar containing liquid nitrogen. For lysis, the following chemicals were added to final concentrations of 8 M urea, 4% CHAPS, 40 mM Tris, 65 mM DTE. If pre-fractionation by SDS PAGE was performed, 400 μM TLCK and 400 μM TCPK protease inhibitors were added.

### SDS PAGE

Prior to SDS-PAGE the samples were mixed with 5× sample buffer. SDS-electrophoresis (overall gel size 7 cm (L) × 8.5 cm (W) × 0.75 mm) was performed using a 1.5 cm 4% stacking gel (0.5 M Tris-HCl pH 6.8, 4% acrylamide-/bis-acrylamide (37.5/1), 0.1% w/v SDS, 0.05% w/v APS, 0.1% v/v TEMED) and a 12% separation gel (1.5 M Tris-HCl pH 8.8, 12% acrylamide/bisacrylamide (37.5/1), 0.1% w/v SDS, 0.05% w/v APS, 0.05% v/v TEMED) with a mini-ProteanTM II device (Bio-Rad, Hercules, USA). Gels were run for 15 min at a constant voltage of 100 V and for additional 60 min at 200 V in SDS running buffer (25 mM Tris, 192 mM glycin, 0.1% w/v SDS). The gels were stained overnight (50% v/v methanol, 0.05% w/v Coomassie brilliant blue R-250, 10% v/v acetic acid) and destained for at least 8 h (5% (v/v) Methanol with 7% (v/v) acetic acid).

### Gel slicing and tryptic in-gel digest

Prior to gel slicing, the gels were washed twice in water. After washing, each gel line was cut into 10 slices using a scalpel. Each slice was transferred in a 1.5 mL reaction tube and equilibrated twice with 50 mM NH_4_HCO_3 _for 10 min. To reduce and block the cystein residues, the gel slices were incubated for 45 min in 50 mM NH_4_HCO_3_/10 mM DTE at 65°C, followed by a 30 min incubation step in 50 mM NH_4_HCO_3 _with 55 mM iodacetamide. Prior to digestion, gel pieces were washed twice for 15 min in 50 mM NH_4_HCO_3 _and minced with a pipette tip. Tryptic hydrolysis was performed overnight at 37°C in 30 μL 50 mM NH_4_HCO_3_with 1 μg porcine trypsin (Promega, Madison, USA) per gel slice. The supernatant was collected and preserved. The peptides were further extracted with 50 μL 50 mM NH_4_HCO_3 _and a subsequent treatment using 50 μL 80% ACN. Both extraction steps were performed for 5 min under sonification (Sonorex RK100, Bandelin, Berlin, Germany). The ACN supernatant and the NH_4_HCO_3 _fractions were combined and concentrated to a volume of 10 μL using a SpeedVac concentrator (Bachover, Vacuum Concentrator). Prior to 2D-LC-MS/MS analysis the peptide were desalted using Pepclean C-18 spin columns (Pierce) as described by the manufacturer.

### 1D-LC separation

The 1D-nano-LC separation was performed on a multi-dimensional liquid chromatography system (Ettan MDLC, GE Healthcare). Peptides were loaded on a RP trap column with a flow-rate of 6 μL per min (Loading buffer: 0.1% formic acid; Trap column: C18 PepMap 100, 5 μm bead size, 300 μm i.d., 5 mm length, LC Packings) and subsequently separated with an analytical column (C18 PepMap 100, 3 μm bead size, 75 μm i.d.; 15 cm length, LC Packings) with a 72 min linear gradient (A: 0.1% formic acid, B: 84% ACN and 0.1% formic acid) at a flow rate of 260 nL/min.

### 2D-LC separation

The 2D-nano-LC separation was performed on a multi-dimensional liquid chromatography system (Ettan MDLC, GE Healthcare). An online salt step configuration was chosen, in which 10 μg of the desalted peptide mixture was injected onto a 50 × 0.32 mm SCX column (BioBasic, Thermo Electron) and eluted at a flow rate of 6 μL/min with 6 discrete salt plugs of increasing salt concentration (10, 25, 50, 100, 500 and 800 mM NH_4_Cl in 0.1% formic acid and 5% ACN). The eluted peptides were bound on a RP trap column (C18 PepMap 100, 5 μm, 300 μm i.d. 5 mm, LC Packings) and subsequently separated on the second-dimension RP column (C18 PepMap 100, 3 μm, 75 μm i.d. 15 cm, LC Packings) with a 72 min linear gradient (A: 0.1% formic acid, B: 84% ACN and 0.1% formic acid) at a flow rate of 260 nL/min.

### Mass spectrometry

Mass spectrometry was performed on a linear ion trap mass spectrometer (Thermo LTQ, Thermo Electron) online coupled to a nano-LC system. For electrospray ionization a distal coated SilicaTip (FS-360-50-15-D-20) and a needle voltage of 1.4 kV was used. The MS method consisted of a cycle combining one full MS scan (Mass range: 300–2000 m/z) with three data dependant MS/MS events (35% collision energy). The dynamic exclusion was set to 30 s.

### Database search and data analysis

The MS/MS data were searched with Mascot Version: 2.1.03 (Matrix Science, Boston, USA) using the following parameters: i) Enzyme: Trypsin, ii) Fixed Modification: Carbamidomethyl (C), iii) Variable modifications: Oxidation (M); iv) Peptide tol. 2 Da, v) MS/MS tol. 0.8 Da, vi) Peptide charge 1+, 2+ and 3+, vii) Instrument ESI-TRAP and viii) Allow up to 1 missed cleavages. Mascot results were further validated with the open source software "Trans-Proteomic Pipeline" (TPP) V3.5 freely available from the Seattle Proteome Center . Therefore the Mascot DAT files were first converted to mzXML, merged and evaluated on the peptide level with the built-in PeptideProphet tool. To generate the list of identified proteins (false positive discovery rate of = 1%) the ProteinProphet tool was used. Furthermore, randomized versions of the applied databases were appended to the original databases using the decoy perl script (Matrix Science, Boston, USA) downloadable at . The number of false positive identifications (randomized sequences) using the Mascot/TPP combination and the corresponding probability thresholds was determined.

### Ontology analysis

Protein entries from the *Daphnia *filtered models database v1.1 were BLASTp-searched  in the Swiss-Prot database . Homologue protein entries (E-values < 0.01) were subjected to ontology analysis using the PANDORA server .

## Abbreviations

1D: one-dimensional; 2D: two-dimensional; DGC: *Daphnia *Genomics Consortium; ESI: electrospray ionization; EST: expressed sequence tag; GO: gene ontology; LC: liquid chromatography; LC: liquid chromatography; MS/MS: tandem mass spectrometry; MS: mass spectrometry; PTMs: post-translational modifications; RP: reversed phase; SCX: strong cation-exchange.

## Authors' contributions

TF participated in the design of the study, performed the LC-MS/MS experiments, data analysis as well as data interpretation and contributed to the writing of the manuscript. CL initiated and coordinated the study and participated in its design; supervised the biological part of the study; performed sample preparation; performed critical reading and writing of the paper. RF and TB carried out *Daphnia *cultivation, preparation of samples for mass spectrometry and contributed to the bioinformatic processing of the data. GJA supervised the proteomic part of the work and contributed to project conception and manuscripts writing.

## Supplementary Material

Additional File 1 **Identified *Daphnia pulex *proteins**. Contains *Daphnia pulex *proteins, ProteinProphet error graph, a list of identified *Daphnia pulex *peptides and proteins with corresponding PeptideProphet, ProteinProphet and Mascot scores and results of BLAST searches. Click here for file

Additional File 2 **Identified *Daphnia longicephala *proteins**. Contains *Daphnia longicephala *proteins, ProteinProphet error graph, a list of identified *Daphnia longicephala *peptides and proteins with corresponding PeptideProphet, ProteinProphet and Mascot scores and results of BLAST searches. Click here for file
